# Protecting or Excluding? Ethical Tensions in Integrated Care Research with People with Complex Needs

**DOI:** 10.5334/ijic.11036

**Published:** 2026-09-01

**Authors:** Catherine Hudon, Marie-Dominique Poirier

**Affiliations:** 1 Department of Family Medicine and Emergency Medicine, Universitéde Sherbrooke, 3001 12e Avenue Nord, J1H 5 N4, Sherbrooke, Quebec, Canada; 2 Patient partner, Canada

**Keywords:** integrated care, ethics, research inclusion, gatekeeping, complex needs, soins intégrés, éthique, recherche inclusive, filtrage des patients, besoins complexes

## Abstract

This perspective paper draws on our experience as a clinician researcher and a patient partner involved in the implementation and evaluation of an integrated care program for people with complex needs. Although an organization actively engaged in integrated care agreed to participate in a research project evaluating implementation, no patient referrals were ultimately received from case managers. Using a real-world vignette, we examine how well-intentioned protective practices within integrated care settings can lead to de facto exclusion from research. We explore the ethical tensions this creates—particularly regarding justice, autonomy, and equity—and outline practical lessons to support more inclusive research practices embedded in integrated care programs.

## Abstract (fr)

Cet article de réflexion s’appuie sur notre expérience de chercheuse clinicienne et de patiente partenaire engagées dans la mise en œuvre et l’évaluation d’un programme de soins intégrés destiné aux personnes ayant des besoins complexes. Bien qu’une organisation de santé fortement engagée dans cette approche ait accepté de participer à un projet de recherche sur sa mise en œuvre, aucun patient n’a finalement été orienté vers nous par les gestionnaires de cas. À partir d’une vignette clinique, nous examinons comment des pratiques de protection bien intentionnées, au sein des structures de soins intégrés, peuvent mener à l’exclusion de certains patients de la recherche. Nous explorons les tensions éthiques qui en découlent — notamment en matière de justice, d’autonomie et d’équité — et présentons des pistes concrètes pour favoriser des pratiques de recherche plus inclusives au sein des programmes de soins intégrés.

## Context and Aim

Mrs Smith, aged 42, has recently experienced eviction and has spent several months living on the streets. She faces multiple physical health issues, including diabetes and chronic obstructive pulmonary disease, alongside mental health challenges and substance dependence. A research project embedded within her hospital’s integrated care program aims to assess patients’ experiences. Mrs Smith meets the eligibility criteria and has agreed to join the program. However, because referrals are coordinated by case managers, and concerned that participation might add to her existing burdens, the case manager decides not to inform her about the study, thereby excluding her from the opportunity to take part.

This scenario reflects challenges encountered during the real-world implementation and evaluation of an integrated care program for people with complex needs, in which the authors—a clinician researcher and a patient partner—were directly involved. Our reflections are grounded in lived experience of program implementation, as well as close collaboration with case managers and service organizations. Despite institutional support for the research, no patient referrals were ultimately made by case managers, prompting critical reflection on the ethical and organizational dynamics at play.

While not uncommon in research settings, this situation raises significant ethical concerns: can protective efforts by professionals aimed at safeguarding individuals with complex needs inadvertently lead to their exclusion from research and result in unintended harm to patients? The aim of this perspective paper is to reflect on how recruitment practices within integrated care settings may unintentionally exclude people with complex needs from research participation, and to identify lessons that can inform more just and inclusive approaches to research embedded in integrated care.

## People with Complex needs in Integrated Care Contexts

People eligible for integrated care programs typically face complex health and social needs, characterized by the accumulation and interaction of physical, mental, and social challenges. Socioeconomic hardship significantly increases the risk of developing both physical and mental health issues [1], resulting in growing numbers of individuals living with interconnected and compounding forms of vulnerability.

For individuals with complex needs, participation in both care and research may be particularly demanding. Multiple factors, individually or in combination, may hinder their involvement, including difficulties with understanding, limited mobility, unstable living conditions, or insufficient resources [2,3]. These barriers can affect their access to information, the capacity to provide informed consent, and the ability to manage the practical aspects of taking part in research.

Within integrated care settings, these challenges are routinely addressed through personalized coordination, flexibility, and the provision of additional support. However, when similar supportive logics are not extended to research participation, exclusion may become the default response—not because individuals are unwilling or incapable, but because systems are not designed to accommodate complexity within research processes.

## Ethical Tensions in Integrated Care Research

The principle of justice requires that individuals should not be excluded from the benefits of research. Researchers, ethics boards, and institutions have a responsibility to ensure fair and equitable inclusion, as well as an appropriate distribution of research benefits. Exclusion from research should not be based on characteristics such as culture, language, gender, race, ethnicity, age, or disability unless there is a valid reason. Inclusion and exclusion criteria must be justified by the research question. Importantly, individuals should not be automatically excluded on the basis of perceived vulnerability or life circumstances [2].

In integrated care models, research is often embedded within service delivery and depends on frontline roles to connect service users with evaluation activities. Case managers commonly occupy a central coordinating position at the interface between service users, care teams, and research processes [4,5]. When access to research is mediated through discretionary professional judgement, a structural ethical tension arises, as decisions about participation may shift away from informed choice and toward selective access, potentially undermining commitments to inclusion, autonomy, and dignity [6]. From an ethical perspective, justice in integrated care research requires more than protection from harm; it also demands that participation remains grounded in informed choice and that the experiences of people with complex needs are not systematically excluded from the knowledge produced.

## Ethical Implications for Integrated Care Practice

Health and social care professionals often develop a protective reflex toward people with complex needs [7,8]. Motivated by a desire to avoid placing an additional burden on individuals already facing significant vulnerability, professionals may, sometimes unconsciously, decide on their behalf not to offer them the opportunity to participate in research.

In our experience, this form of protective gatekeeping occurred despite the explicit agreement of an integrated care organization to participate in a research project evaluating program implementation [9]. As a result, eligible service users were not informed about the study and were deprived of both the freedom to choose and the opportunity to contribute their perspectives to research intended to improve integrated care [10].

This situation highlights a fundamental tension within integrated care practice: approaches designed to protect, support, and coordinate care may, if left unexamined, undermine the principles of inclusion and person-centredness that integrated care seeks to promote. Although well-intentioned, such mechanisms risk perpetuating the invisibility of people with complex needs in the evidence base used to inform integrated care design, policy, and improvement.

Finding an appropriate balance between protection and inclusion is therefore essential to safeguarding both safety and autonomy. Achieving this balance requires not only individual reflexivity among professionals, but also organizational and ethical attention to how research is embedded within care delivery. Ethics committees and organizations implementing integrated care must consider how governance structures, safeguards, and recruitment processes may inadvertently reinforce exclusion when applied without flexibility or support.

## Key Lessons for Integrated Care Practices

Restoring and sustaining a balance between protection and inclusion in integrated care research requires renewed emphasis on respecting service users’ autonomy and right to choose. Rather than making assumptions on their behalf, researchers and integrated care professionals should prioritize creating conditions that enable informed choice and support self-determination.

Drawing on our experience as clinician researcher and patient partner, several practical lessons emerge for conducting research in integrated care programs:

- Opportunities to participate in research should be systematically offered to eligible service users, rather than filtered through discretionary gatekeeping.
- Additional support—such as flexible consent processes, health-literacy-sensitive communication strategies, or accompaniment—should be developed for patients to enable participation instead of default exclusion.
- Integrated care teams should be encouraged to critically reflect on protective practices and their unintended consequences for equity and knowledge production.
- Possibility of implicit exclusion from research embedded within care delivery should be made visible and discussed within integrated care governance structures.

By actively addressing these issues, integrated care programs leaders can ensure that research practices align with values of inclusion, equity, and person-centred care, while generating evidence that more accurately reflects the lived experiences of people with complex needs.
